# Comparative analysis of the spatio-temporal dynamics of rotifer community structure based on taxonomic indices and functional groups in two subtropical lakes

**DOI:** 10.1038/s41598-017-00666-y

**Published:** 2017-04-03

**Authors:** Xinli Wen, Pan Zhai, Ruonan Feng, Ruijie Yang, Yilong Xi

**Affiliations:** 1 Key Laboratory of Biotic Environment and Ecological Safety in Anhui Province; College of Life Sciences, Anhui Normal University, Wuhu, 241000 China; 2 Collaborative Innovation Center of Recovery and Reconstruction of Degraded Ecosystem in Wanjiang City Belt, Anhui Province, Wuhu, 241000 China

## Abstract

Little research has focused on how rotifer communities respond to eutrophication based on their combined taxonomic and functional indices. In this research, the relationship of the environment and rotifer communities was comparatively investigated in two subtropical lakes over one year. The taxon-based indices, including species number (*S*), Margalef index (*D*), Simpson index (*d*), Shannon-wiener index (*H*′), and functional traits relying on the guild ratio (GR) and the modified guild ratio (GR′) from the moderately eutrophic Lake Xiyanghu were significantly lower than those from the slightly eutrophic Lake Jinghu. Redundancy analysis (RDA) showed that both lakes were distinct from each other. Taken together, the findings indicate that trophic state was an important factor affecting rotifer community structure. In addition, the average annual GR′ of Lake Xiyanghu was <0, suggesting the dominancy of microphagous rotifers. Over time, *S*, *D*, *d*, and *H*′ were positively correlated with temperature and phosphorus levels in Lake Jinghu, but were negatively correlated with NH_4_^+^-N levels in Lake Xiyanghu. Only GR′ was negatively associated with chlorophyll-*a* in Lake Xiyanghu, implying that the functional index (GR′) might be an effective tool to explore the relationship between trophic state and the rotifer community in seriously eutrophic lakes.

## Introduction

Eutrophication is still considered to be the most pressing water quality problem in both fresh and salt waters^1^. In lake water, eutrophication refers to changes in water chemical properties triggered by an over accumulation of nutrients like nitrogen and phosphorus. In China, nearly 60–70% of freshwater lakes are distributed along the eastern coast or along the middle to lower reaches of the Yangtze River. A large majority of these are shallow^2^ and have become increasingly eutrophic since the late 1980s^3, 4^. Rotifers are an important constituent of zooplankton in the aquatic food web and are often dominant in lentic water, such as lakes^5, 6^. Pelagic rotifers are very sensitive to environmental stress and have been shown to respond to pollution^7, 8^ and changes in trophic state^9^. They have been suggested to be excellent targets for ecological water quality monitoring^10^ and for the assessment of the effects of ecological remediation efforts on eutrophic water^11^.

Up until now, the link between the degree of eutrophication and the taxonomic indices of rotifer communities, including species numbers, diversity, evenness indices, and total abundance, has received the most attention in the literature and has thus been well studied and documented^11, 12^. Based on contemporary comparisons of rotifer communities across multiple lakes, many researchers found that, in relation to spatial scales, taxon-based indicators like species richness and diversity tend to decline with increasing trophic levels^13–16^. However, some researchers have suggested that the role of the trophic state on zooplankton communities, including rotifers, has not yet been clearly demonstrated^17^.

Recently, the focus of zooplankton community ecology has shifted from taxon-based indices to function-based research^18^. The use of functional traits has been proposed to be a more effective method of linking community structure to ecosystem function^19^. Functional diversity has been widely investigated and applied to studies of various types of aquatic biology, such as phytoplankton^20^, crustaceans^18, 21^, and copepods^22^. As has been suggested by previous studies^23, 24^, the trait-based indices of GR and GR′ have also been applied in rotifer community ecology, based on the feeding strategies and diets of the rotifer communities^24–27^. The functional diversity of crustacean zooplankton communities in 18 lakes in Canada experienced a linear decline over a TP gradient (as an index of trophic status), while species richness exhibited a unimodal relationship along the same gradient^18^. This indicates that trophic state might also be an important condition affecting functional crustacean zooplankter traits, including cladoceran and copepods. To the best of our knowledge, it is still unknown whether trait-based indices of rotifer GR and GR′ are different in lakes with distinctly different trophic states.

Many studies have focused on the temporal variation in rotifer community structure based on taxon-based indices^11, 12, 28^, but research focused on functional groups has received less attention. Obertegger & Mancan^29^ conducted a case study of the change in GR′ over time, concluding that, compared with the smooth changes observed in taxon-based indices, a dramatic GR′ response to trophic state in rotifer assemblages was found in a deep subalpine lake, Lago Maggiore. This implied that function-based parameters are more sensitive to trophic degree than are taxon-based indices, despite the fact that taxon-based indices can still be used as biological indicators for water quality and eutrophication. Nevertheless, water temperature, nutrient content, and other environmental variables fluctuate considerably with changing seasons in subtropical lakes, directly influencing prevalence of different rotifer species and seasonal succession^9, 30^. Accordingly, the effects of trophic state, water temperature, and other environmental variables on the functional traits of rotifer communities in subtropical lakes deserve further investigation.

In this report, a comparative study was performed addressing the temporal and spatial patterns of rotifer communities based on taxon indices and functional groups between two lakes with slightly different trophic states. The aims of the study are as follows: (a) to test the hypothesis that trophic state is an indispensable factor affecting the rotifer community structure based on taxonomic indices and (b) to assess the susceptibilities of taxon-based indices and rotifer functional groups to environmental factors, including trophic state.

## Materials and Methods

### Study sites

Lake Jinghu and Lake Xiyanghu are both shallow lakes (average depth of 1.5 m) and are situated in the center of Wuhu city (31.21°N, 118.22°E), located near the middle and lower reaches of the Yangtze River, and composed of two connective lake regions (Supplementary Fig. S1). They have a high societal value by providing recreational opportunities and aesthetic benefits to residents, with open water areas of about 150000 and 50000 m^2^, respectively. In Lake Xiyanghu, the submerged and emerged macrophytes cover a part of the basin and the phytoplankton is dominated by Euglenoides in the late spring and Cyanobacteria in the summer and early autumn.

### Rotifer sampling

The sampling was performed between July 2012 and June 2013 at two sites for each lake. Rotifer samples were collected twice a month at approximately the same time. On each sampling date, a quantitative sample was obtained by filtering 15 L of integrated lake water (5.0 L water at each depth from surface to bottom at 0.5 m intervals was collected using a 2.5 L organic glass water sampler) through a plankton net (mesh size 25 μm) at each station. The retained rotifers were preserved in 4% sucrose formaldehyde in the laboratory and were then concentrated to 30 ml for a minimum of 48 h. Rotifers were examined with at least three Sedgewick-Rafter sub-samples under an Olympus BH-2 microscope at 100× magnification^31^. Qualitative samples for identification of rotifer species were collected by filtering 40 L lake water through a 64 μm nylon sieve. The rotifer densities were then determined for discrete species, whereupon species identification was carried out on fixed samples combined with living materials, as previously described in rotifer taxonomy references^32, 33^.

### Environmental conditions

Surface water temperature was measured during sampling with a mercury thermometer, the pH value was determined with an HI-8424 acidometer (Hanna, Italy), and the water transparency was recorded using a secchi-disk (SD). In order to quantify chlorophyll-*a* (Chl-*a*) content, 1.0 L water from a 10.0 L pooled sample was filtered through Whatman GF/C glass-fiber filters (1.2 μm pore size), and was extracted with 90% acetone overnight in the dark, after fully grinding the filters in a mortar. Chl-*a* concentrations were determined spectrophotometrically and calculated according to the method described by Lorenzen^34^. The contents of total nitrogen (TN), nitrate nitrogen (NO_3_^−^-N), ammonia nitrogen (NH_4_^+^-N), total phosphorus (TP), and PO_4_^3−^ were measured once a month according to Huang’s methods^35^. The comprehensive trophic state index (TSI) was used to assess the trophic state of both lakes, and was calculated with the equations described by Carlson and OECD^36, 37^.

### Analyses of taxon- and trait-based indices

The Shannon–Wiener (*H*′), evenness (*E*), and Margalef (*D*) indices were used to estimate changes in biodiversity, with the formula *H*′ = −*P*_*i*_Σlog_2_ *P*_*i*_, *E* = *H*/*H*_*max*_, *D* = (*S* − 1)/log_2_ *N*, where *P*_*i*_ is the density percentage of the *i* ^th^ species, and *S* and *N* are the total species number and total number in a sample, respectively. The density of the dominant species was confirmed by dominance grade (*Y*), which was calculated as *Y* = (*N*_*i*_/*N*) × *fi*, where *N*_*i*_ and *N* indicate the density of the *i* ^th^ species and the total rotifer density in a community, respectively, and *fi* represents the occurrence frequency of *i* ^th^ species among annual samples in a lake.

Two trait-based indicators were selected for use in this study^23, 24^. These were the guild ratio (GR), defined as the ratio of raptorial to microphagous species, and a modified form of the GR (i.e., GR′), which is calculated as (biomass raptorial − biomass microphagous)/(total rotifer biomass). The estimates of rotifer biomass were calculated by applying biovolume equations based on their body length^38^, which was measured under a microscope.

### The effects of environmental variables on taxon-based indices and functional traits

A paired-samples t-test was used for statistical analysis to detect differences between environmental variables. The taxon- and functional indices that were measured were compared between the two stations in the same lake and between the two lakes.

Canonical correspondence analysis (CCA) was carried out using CANOCO 4.5 (SCIENTIA Software), in order to define the classification of the rotifer communities between the two lakes, as well as to determine the relationships between environmental variables and the rotifer data matrix based on the taxonomic indices^39^. Species composition data were log (x + 1) transformed and environmental variables were kept standardized. Either CCA or the Redundancy Analysis (RDA) procedures were selected depending on the length of the gradient from the preliminary analysis by means of detrended correspondence analysis (DCA)^39–41^. Only the explanatory variables displaying significant and varying inflation factors less than 10 (VIF<10) were included in this study (Monte Carlo permutation test with 499 permutations, α = 0.05)^39, 41, 42^.

Pearson’s correlation was carried out between the taxon-based indices [including species number (*S*), Margalef index (*D*), Shannon–Wiener index (*H*′) and Pielou evenness index (*J*)] and environmental variables with SPSS 11.5 software. SPSS was also used to analyze trait-based indices (GR and GR′) in relation to environmental factors. Environmental variables that displayed significant correlations were selected for evaluation of their independent contribution to levels of taxon-based indices and functional traits. To this end, multiple regression analysis was used to compensate for covariance, using the forward stepwise selection procedure to select the variables with a significant *F*-value that significantly increased the regression sum of the squares.

## Results

### Physicochemical variables and eutrophication levels

The annual mean TSI values in Lake Jinghu and Lake Xiyanghu were 51.00 and 63.24, respectively, characterizing Lake Jinghu as slightly eutrophic and Lake Xiyanghu as moderately eutrophic, according to the standards set forth by Cai^43^.

Similar temporal variation trends were found, but with no discernable differences in environmental factors between the two stations in each lake (paired t-test, *p* > 0.05). Therefore, the average value of the environmental factors in the two stations per lake was used to depict the seasonal dynamics of the individual lake and to comparatively analyze the differences between both lakes across the span of one year. The annual values of all of these measured conditions are listed in Table 1.

**Table 1:** Annual average values of physico-chemical factors and TSI (±SE) in Lake Jinghu and Lake Xiyanghu.

Environmental parameters	Lake Jinghu	Like Xiyanghu
Water temperature (WT) (°C)	18.87 ± 1.90	19.00 ± 1.98
pH Value	8.26 ± 0.08	8.45 ± 0.10
Secchi-disk (SD) (m)	0.77 ± 0.015*	0.37 ± 0.03*
Chlorophyll-*a* concentration (µg/L)	58.57 ± 2.87*	188.52 ± 25.81*
TP (mg/L)	0.07 ± 0.007*	0.41 ± 0.04*
PO_4_^3−^ (mg/L)	0.04 ± 0.007*	0.23 ± 0.03*
TN (mg/L)	0.83 ± 0.08*	7.34 ± 1.95*
NO_3_^−^-N (mg/L)	0.06 ± 0.03*	0.52 ± 0.11*
NH_4_^+^-N (mg/L)	0.07 ± 0.02*	0.88 ± 0.30*
Comprehensive trophic state index (TSI)	51.00 ± 0.73*	63.24 ± 1.54*

(*P < 0.05).

**Figure 1:**
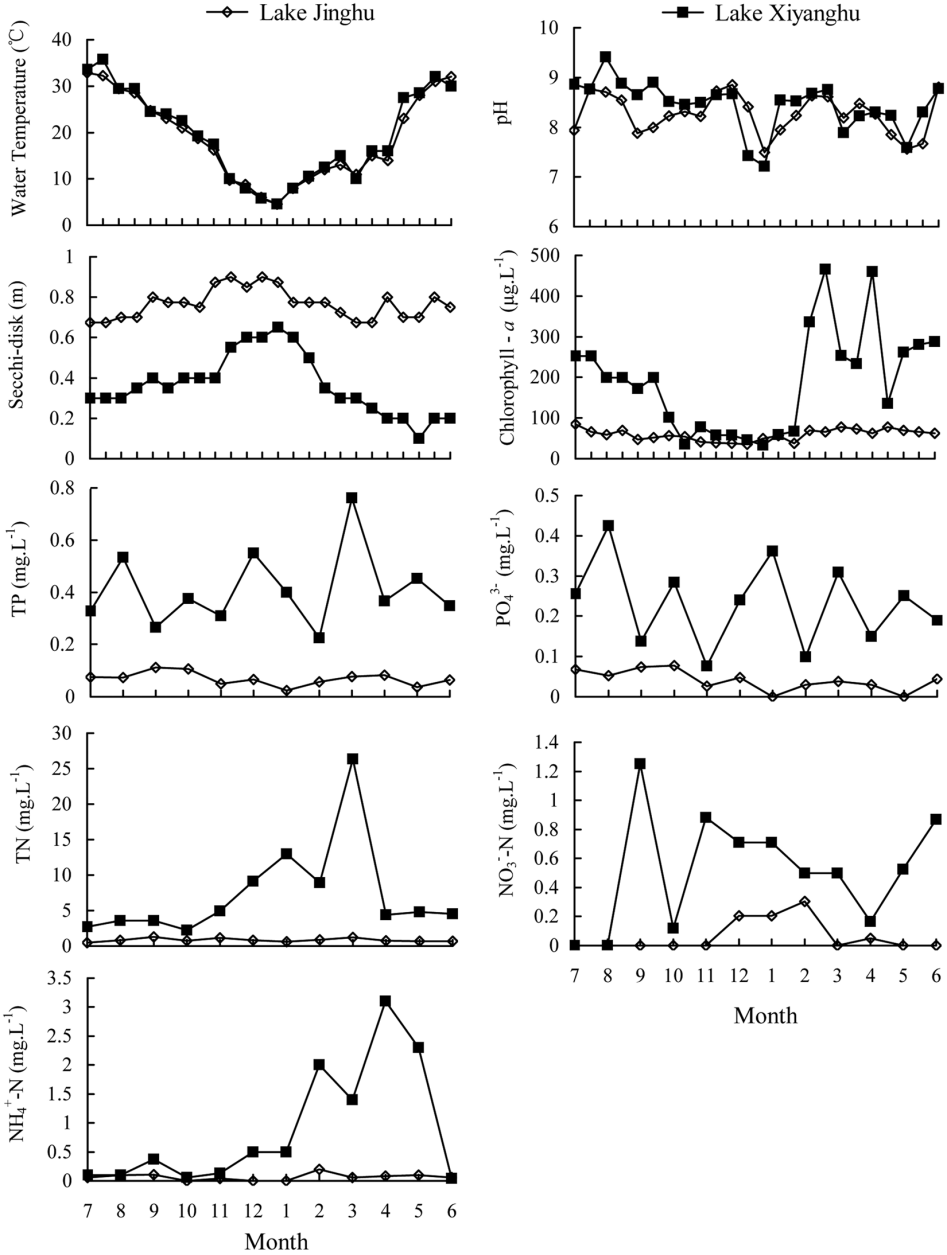
Annual dynamics of physico-chemical factors in Lake Jinghu and Lake Xiyanghu.

Both lakes showed clear seasonal variations in water temperature (WT) and secchi-disk (SD) clarity readings, with WT ranging from 4.5 °C to 33.5 °C in both lakes and SD ranging between 0.67–0.87 m in Lake Jinghu and 0.2–0.7 m in Lake Xiyanghu. The pH value showed relatively smooth changes, ranging between 7.5 and 8.5 in both lakes, except for an August peak of 9.5 in Lake Xiyanghu. Lake Xiyanghu also showed dramatically seasonal variations in Chl-*a*, TN, NO_3_^−^-N, NH_4_^+^-N, TP, and PO_4_^3−^ concentrations in contrast with values seen in Lake Jinghu (Fig. 1). In both lakes, the Chl-*a* concentration was higher in the spring (March–May) and summer (June–August) than was seen in the autumn (September–November) and winter (December–February). TN, TP, and PO_4_^3−^ concentrations were higher in the autumn in Lake Jinghu. However, TN, TP, and NH_4_^+^-N were shown to be present in higher concentrations in the winter and spring in Lake Xiyanghu (Fig. 1).

Significant differences in the measured physicochemical conditions were seen aside from WT and pH (Paired t-test, *p* < 0.05; Table 1). The SD findings in Lake Jinghu were obviously lower than those in Lake Xiyanghu (Paired t-test, *p* < 0.05, Table 1), but other environmental variables measured in this study were markedly higher for Lake Jinghu than for those in Lake Xiyanghu (Paired t-test, *p* < 0.05; Table 1).

### Variations in rotifer community structure based on taxon-based indices

A total of 46 rotifer species, belonging to 16 families and 22 genera, were observed in Lake Jinghu and 47 species from 15 families and 20 genera were similarly found in Lake Xiyanghu. *Brachionus*, *Trichocerca*, and *Filinia* had the highest species numbers in both lakes, accounting for 13.04%, 10.87%, and 8.70% of the total rotifer species in Lake Jinghu, and 14.89%, 10.64%, and 10.64% of the total rotifer species in Lake Xiyanghu (Supplementary Table S1).

Lake Jinghu showed an obvious annual dominance of *Poyarthra vulgaris* and *Anuraeopsis fissa*, followed by *Rhinoglena frontalis*, *Brachionus angularis*, and *Trichocerca pusilla*, with an annual average density of 24.3, 15.4, 12.9, 11.1, and 10.8 ind./L, respectively. In Lake Xiyanghu, the prevailing rotifer species were *P*. *vulgaris*, *Brachionus calyciflorus*, and *B*. *angularis*, followed by *T*. *pusilla* and *Filinia longiseta*, whose average density was 59.3, 39.7, 39.0, 27.9, and 27.4 ind./L, respectively. The monthly predominant species was *P*. *vulgaris* in both lakes except for December 2012 and January 2013 in Lake Jinghu and February and March 2013 in Lake Xiyanghu, when *R*. *frontalis* and *B. calyciflorus* were dominant, respectively.

**Figure 2:**
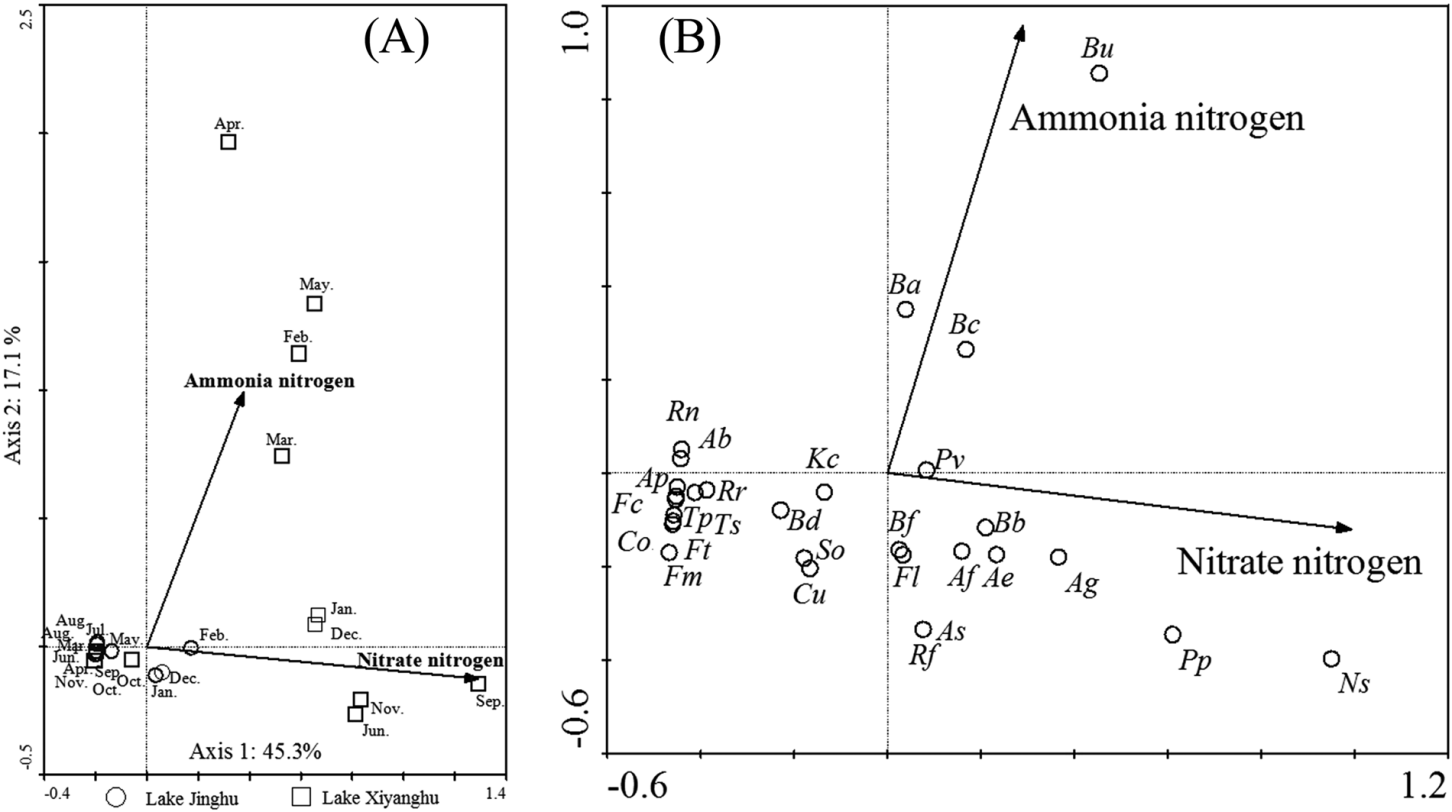
RDA ordination biplots of the site scores (**A**), species composition (**B**), and selected environmental variables in Lake Jinghu and Lake Xiyanghu. (Ammonia nitrogen = NH_4_^+^-N; Nitrate nitrogen = NO_3_^−^-N).

Paired t-tests indicated that species numbers (*S*), the Margalef index (*D*), Simpson index (*d*), and Shannon-wiener index (*H*′) were significantly lower for the rotifer community in Lake Xiyanghu than those recorded in Lake Jinghu (*p* < 0.05; Table 2), but the Pielou evenness index (*J*) was remarkably higher in Lake Xiyanghu than in Lake Jinghu (*p* > 0.05; Table 2). Based on results from the Monte Carlo permutation test and the VIF < 10, only NH_4_^+^-N (VIF = 1.04) and NO_3_^−^-N (VIF = 1.04) were selected for the redundancy analysis (RDA), which showed that the first two ordination axes explained 62.4% of the species-environment variability (Fig. 2). NO_3_^−^-N and NH_4_^+^-N were shown to be positively correlated with axis1 and axis2, respectively. The diagram distinctly shows that Lake Jinghu has a noticeably different rotifer community structure than does Lake Xiyanghu (Fig. 2A). The species-environment relationship is presented in Fig. 2B. Only *B*. *calyciflorus*, *B*. *angularis*, and *B*. *urceolaris* were positively associated with NH_4_^+^-N, but *R*. *frontalis*, *B*. *forficula*, *F*. *longiseta*, and *A*. *saltans* were inversely correlated with NH_4_^+^-N. *P*. *vulgaris*, *B*. *budapestinensis*, *A*. *fissa*, *A*. *ecaudis*, *A*. *girodi*, *P*. *parasitica*, and *N*. *aurita* were positively correlated with a high level of NO_3_^−^-N.

**Table 2:** Annual average values of taxon-based indices (±SE) [species number (*S*), Simpon index (*d*), Margalef index (*D*), Shannon-Wiener index, (*H*′) Pielou evenness index (*J*)] and trait-based indices (GR and GR′) of the rotifer community in Lake Jinghu and Lake Xiyanghu.

Index	Lake Jinghu	Lake Xiyanghu
Species numbers (*S*)	12.63 ± 0.52**	9.08 ± 0.66**
Simpson index (*d*)	0.77 ± 0.013*	0.74 ± 0.018*
Margalef index (*D*)	2.63 ± 0.08**	2.08 ± 0.11**
Shannon-Wiener (*H*′)	2.62 ± 0.07**	2.32 ± 0.10**
Pielou evenness (*J*)	1.05 ± 0.02**	1.15 ± 0.02**
GR	4.44 ± 0.97*	2.13 ± 0.47*
GR′	0.21 ± 0.07**	−0.18 ± 0.09**

(*p < 0.05; **p < 0.01).

The species numbers and the Margalef, Simpson, and Shannon-wiener indices tended to decrease during winter and spring (December 2012 and April 2013) in both lakes, but were more obvious in Lake Xiyanghu (Supplementary Fig. S2). On the other hand, the species numbers and the Pielou evenness, Margalef, Simpson, and Shannon-wiener indices of the Lake Jinghu rotifer community were positively correlated with water temperature and PO_4_^3−^ (*p* < 0.05; Table 3). Species numbers and the Shannon-wiener index were also positively correlated with TP (Table 3). In Lake Xiyanghu, species numbers, and the Margalef, Simpson, and Shannon-wiener indices were all positively correlated with water temperature and cladocera density, but were negatively associated with NH_4_^+^-N (*p* < 0.05; Table 3). Multiple regression models revealed that NH_4_^+^-N was more significantly correlated with species numbers, and Margalef, Simpson, and Shannon-wiener indices than were the excluded variables of water temperature and cladocera density (*p* > 0.05; Table 4).

**Table 3:** Pearson moment correlation between taxonomic indices (species numbers = *S*, Simpson index = *d*, Margalef index = *D*, Shannon-Wiener index = *H*′, Pielou evenness index = *J*) and each environmental factor (WT = water temperature, SD = Secchi disc, Chl-*a* = Chlorophyll-*a*) in Lake Jinghu and Lake Xiyanghu.

Environmental factors	Lake Jinghu					Lake Xiyanghu				
	*S*	*d*	*D*	*H*′	*J*	*S*	*d*	*D*	*H*′	*J*
WT (°C)	0.44**	0.53**	0.33*	0.60**	0.44**	0.61**	0.48**	0.44**	0.53**	−0.23
pH	0.02	0.16	0.12	0.19	0.28	0.14	0.12	0.09	0.17	0.09
SD (m)	−0.20	−0.18	0.07	−0.21	−0.15	−0.27	−0.14	0.06	−0.15	0.17
Chl-a (µg/L)	−0.05	0.05	−0.24	0.004	0.06	−0.05	−0.09	−0.19	−0.14	−0.12
TP (mg/L)	0.68*	0.52	0.56	0.60*	0.40	−0.20	−0.13	−0.02	−0.14	0.11
PO_4_^3−^ (mg/L)	0.73**	0.75**	0.65**	0.82**	0.64*	0.04	0.41	0.32	0.39	0.53
TN (mg/L)	0.003	0.19	0.04	0.20	0.23	−0.49	−0.25	−0.09	−0.40	0.30
NO_3_^−^-N (mg/L)	−0.07	−0.38	0.11	−0.38	−0.44	−0.02	−0.05	0.03	−0.12	−0.21
NH_4_^+^-N (mg/L)	−0.09	−0.39	−0.15	−0.28	−0.29	−0.70**	−0.72**	−0.76**	−0.77**	−0.11
Cladocera (ind./L)	0.15	0.17	0.06	0.24	0.20	0.41*	0.36*	0.37*	0.40*	0.02
Copepoda (ind./L)	0.27	0.25	0.21	0.28	0.16	0.17	0.23	0.06	0.17	0.09
Naupii (ind./L)	0.20	0.18	0.10	0.23	0.13	0.14	0.24	0.03	0.10	0.07

(* *p* < 0.05; **p < 0.01).

**Table 4:** Forward stepwise regression analyses between dependent variables (Y) of *S*, *D*, *d*, and *H*′ and independent variables of the unique included environmental factors of NH_4_^+^-N.

Taxonomic indices	Full model	R^2^	P	Standardized coefficient
Species numbers (*S*)	Y = −2.70X_NH4+_ + 10.47	0.49**	<0.05	−0.70*
Simpson index (*d*)	Y = −0.09X _NH4+_ + 0.81	0.52**	<0.01	−0.72**
Margalef index (*D*)	Y = −0.44X _NH4+_ + 2.69	0.57**	<0.01	−0.76**
Shannon-Wiener (*H*′)	Y = −0.45X _NH4+_ + 2.71	0.59**	<0.01	−0.77**

Standardized coefficient from this multiple regression analyses represents the effect intensity of the included the environmental factors on dependent variables such as *S*, *D*, *d*, and *H*′ in the full model (*p < 0.05; **p < 0.01).

### Variations in the functional traits of rotifer communities

Paired t-tests indicated that the functional parameters of rotifer community GR and GR′ in Lake Xiyanghu were significantly lower than those determined for Lake Jinghu (*p* < 0.05; Table 2). In particular, a negative annual GR′ average was found in the rotifer community of Lake Xiyanghu (Table 2).

The seasonal dynamics of GR and GR′ are shown in Supplementary Fig. S3. Generally, the GR in Lake Jinghu showed a relatively low value from December 2012 to early April 2013 and from late July to early September 2012, as well as during January and June 2013 in Lake Xiyanghu. Similarly, GR′ presented with a negative value from late December 2012 to early April 2013 in Lake Jinghu, and from late July to early September 2012 and during January and May 2013 in Lake Xiyanghu.

**Figure 3:**
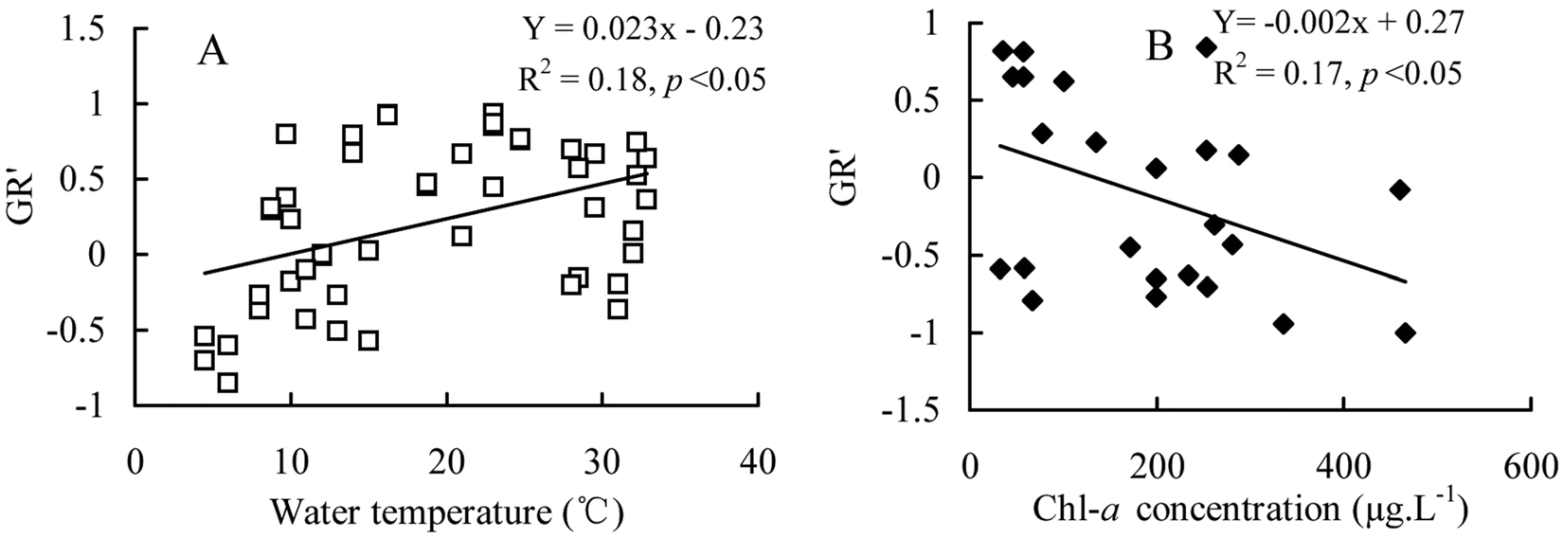
Relationships between GR′ and water temperature as well as Chl-*a* concentration. (**A**: Lake Jinghu, **B**: Lake Xiyanghu).

Pearson moment correlation and regression analyses indicated that only GR′ was positively correlated with the unique factor of water temperature in Lake Jinghu (*p* < 0.05; Table 5; Fig. 3), but was negatively associated with Chl-*a* concentration in Lake Xiyanghu (*p* < 0.05; Table 5; Fig. 3). However, GR was not significantly related to any of the environmental variables measured in this study (*p* < 0.05; Table 5).

**Table 5:** Pearson moment correlation between functional indices (GR = the guild ratio, GR′ = the modified guild ratio) and each environmental factor (WT = water temperature, SD = Secchi disc, Chl-*a* = Chlorophyll-*a*).

Environmental factors	Lake Jinghu		Lake Xiyanghu	
	GR	GR′	GR	GR′
WT (°C)	0.13	0.42*	0.02	0.14
pH	−0.13	0.07	0.11	0.006
SD (m)	0.13	−0.09	0.26	0.18
Chl-*a* (µg/L)	−0.16	−0.04	−0.35	−0.42*
TP (mg/L)	−0.18	0.23	0.01	−0.15
PO_4_^3−^ (mg/L)	−0.13	0.38	0.07	−0.06
TN (mg/L)	0.35	0.19	−0.23	−0.47
NO_3_^−^-N (mg/L)	−0.36	−0.37	−0.29	−0.15
NH_4_^−^-N (mg/L)	−0.03	0.18	−0.34	−0.37
Cladocera (ind./L)	0.06	0.28	0.03	0.09
Copepoda (ind./L)	0.06	0.19	0.31	0.11
Naupii (ind./L)	0.07	0.24	0.34	0.14

*p < 0.05; **p < 0.01.

## Discussion

The effects of eutrophication caused by anthropogenic activity in aquatic systems have received much attention over the last decades. Since rotifers undergo parthenogenetic reproduction, population sizes are generally large^44, 45^. In fact, rotifers are believed to be the most dominant zooplankton group in many lentic systems, and their spatial-temporal dynamics are influenced by eutrophication level^12, 13, 16^. Rotifer occurrence is commonly thought to be associated with trophic state^7, 10, 16, 46, 47^. *Brachionus* spp. and *A*. *fissa* are regarded as eutrophic species, while *T*. *pusilla* and *F*. *longiseta* occur in mesotrophic lakes, and *Poyarthra* often dominates in waters with high trophic states^48^. In the present study, rotifers that serve as indicators of eutrophic water were observed in both Lake Jinghu and Lake Xiyanghu, which differ in eutrophication level, implying that species composition of rotifer communities might not be a perfect bioindicator of water quality.

Regulation of rotifer community dynamics has been ascribed to abiotic factors, visibility, water temperature, pH, and nutrients, as well as biotic factors such as food supply, competition with cladocera for food, and predation by copepoda^45^. Previous studies suggested that the trophic state determines the spatial patterns of the individual rotifer community, whereas water temperature controls the seasonal dynamics of the assemblages^16, 49^. In our study, although the densities of potential rotifer competitors and predators were higher in Lake Xiyanghu, the bioplot of RDA with rotifer densities and both abiotic and biotic factors showed that all of the samples from the slightly eutrophic lake (Lake Jinghu) were separated from those of the moderately eutrophic lake (Lake Xiyanghu), indicating that trophic state was important in regulating the spatial dynamics of the surveyed rotifer communities. Nevertheless, the temporal pattern of rotifer communities in the present study was not principally impacted by water temperature, but rather by nitrogenous nutrients.

Biodiversity is one of the most essential characteristics of the aquatic ecosystem for maintenance of stability and as a means of coping with any environmental changes. In the present study, the species diversity (species numbers, Margalef index, Simpson index, and Shannon-wiener index) found in Lake Xiyanghu was significantly lower than those found in Lake Jinghu. Despite the potential pressures of competition and predation from cladocera and copepoda, respectively, relatively serious eutrophication might be the reason for the decreasing diversity of rotifer species in Lake Xiyanghu, since the effects of these planktonic crustaceans were eliminated in RDA results, which was in agreement with the conclusion that species diversity is negatively associated with trophic state^15, 16^.

Rotifer communities have been shown to respond quickly to a wide range of environmental stresses, but most of the studies on the relationship between rotifer groups and the trophic state of lakes have been based on contemporary comparisons of rotifer communities across multiple lakes, rather than on changes in the same lake over time^12^. In our study, the metrics of species diversity, such as species numbers, the Margalef index, Simpson index, and Shannon-wiener index, tended to increase in the summer and autumn and were correlated with water temperature in both lakes, indicating that stenotherm rotifers were generally more prominent in cold water. In Lake Jinghu, the TP was between 0.02 and 0.11 mg.L^−1^, and indices of species diversity showed a positive correlation with phosphorus concentration (TP and/or PO_4_^3−^). The ecological effects of phosphorus on rotifer zooplankton species diversity are still debatable, and have been focused on identifying a positive (productivity increases diversity) or unimodal relationship between the two (low diversity at the lower and upper extremes of productivity)^50–53^. Our findings were in agreement with the established result that zooplankton diversity in reservoirs is positively correlated with TP and that productive environments (natural lakes) gather more species^54^.

However, the key factor affecting the species diversity in the rotifer community of Lake Xiyanghu was found to be NH_4_^+^-N, rather than phosphorus. It is well known that NH_4_^+^ (ionized ammonia) and NH_3_.H_2_O (un-ionized ammonia) are the main manifestations of ammonia nitrogen in fresh bodies of water. Un-ionized ammonia is always harmful for rotifers^55, 56^. In fact, the growth and reproduction of *B*. *calyciflorus* is inhibited with the introduction of un-ionized ammonia in concentrations exceeding 2.57 mg.L^−1^ in a culture media^55^. In Lake Xiyanghu, NH_4_^+^-N showed a higher concentration in the winter and spring and reached a peak of 3.1 mg.L^−1^, which likely reduced the species diversity of the rotifer assemblages in Lake Xiyanghu.

Functional traits are integrative characteristics based on morphological, physiological, or behavioral features of the organism that, when placed in a community context, should provide information on species interactions within food webs and their environment, including feedbacks to ecosystem function^57^. An index of GR′ dependent on the feeding strategy of rotifers ranged from −1 to 1, and was employed in order to study the influence of varying environmental conditions on the rotifer community. A GR′ < 0 indicates dominance of microphagous species, while raptorial rotifers are shown to prevail when GR′ > 0^24^. Microphagous rotifers were generally observed with smaller volumes than were raptorial species, as previously reported^29^. Accordingly, microphagous species may be better adapted to food-poor conditions, since body size is positively correlated with food threshold level^58^. Additionally, small rotifers can more effectively escape from carnivorous invertebrates like copepods^59^. In the present study, the annual average value of GR′ in Lake Xiyanghu was not only remarkably lower than that in Lake Jinghu, but was also less than zero, suggesting that microphagous rotifers were dominant in Lake Xiyanghu. Similarly, some small rotifer species dominated in the Chinese subtropical Lake Donghu, which has an increasingly eutrophic state^28^. Therefore, the predominant microphagous rotifers that were observed in Lake Xiyanghu might be partly due to the higher eutrophication level.

Seasonal dynamics demonstrated that GR was not correlated with any environmental variables. In comparison, GR′ displayed a significant correlation with environmental factors in both lakes, suggesting that GR′ might be more useful than GR for elucidating the associations between environmental variables and the rotifer community. In Lake Xiyanghu, GR′ was negatively correlated with Chl-*a* concentration, the presence of which is an effective indicator for evaluating the level of eutrophication, as well as the index linking phytoplankton biomass with nutrient loading in a given lake with a high trophic status^60^. Higher Chl-*a* concentrations were found during spring and early autumn, while Euglenoides and Cyanobacteria dominated the phytoplankton composition (unpublished data), which are not always favorable for rotifer food^25^. Microphagous rotifers with a relatively small body mass would be more likely to thrive in conditions of poor food quality than would raptorial species, which is an explanation for the negative relationship between GR′ and Chl-*a* concentration in Lake Xiyanghu. In Lake Jinghu, Euglenoides and Cyanobacteria were not dominant^16^, and GR′ was positively related to water temperature. In lakes without serious eutrophication levels, food resources might be the greatest dynamic for rotifer composition, while water temperature might be the key factor driving the seasonal dynamics of the functional groups in the rotifer community.

Rotifers have been suggested to be excellent model organisms for ecological assessment, water quality monitoring, and eutrophication level. Hence, studies on how rotifer communities respond to eutrophication based on their taxonomic and functional indices are of great interest. However, to the best of our knowledge, numerous studies on the relationship between environment and rotifer communities have focused on the spatio-temporal dynamics based on the taxon-based indices, but few studies have focused on the functional traits of rotifer communities. Even fewer have compared the susceptibility of taxon-based indices to environmental factors (e.g., trophic state) with that of functional groups. In the present study, in relation to seasonality, GR′ was positively correlated with water temperature in Lake Jinghu, but was negatively associated with Chl-*a* concentration in Lake Xiyanghu. In comparison, GR was not significantly related to any of the environmental variables, suggesting that it could serve as a more suitable index in the environment-rotifer community relationship than GR. Moreover, the four taxonomic indices were positively correlated with temperature and phosphorus in Lake Jinghu, but were negatively correlated with NH_4_^+^-N in Lake Xiyanghu. GR′ was negatively correlated with Chl-*a* concentration, while the four taxonomic indices negatively correlated with NH_4_^+^-N in Lake Xiyanghu. Those results probably suggest that the GR′ function index is a better tool to analyze the relationship between trophic state and the local rotifer community in eutrophic lakes, as Chl-*a* concentration is a more effective indicator for evaluating the level of eutrophication than NH_4_^+^-N.

Taken together, the present study raised a hypothesis that GR′ is a more effective indicator to assess the responses of rotifers to eutrophication. To confirm this hypothesis, more data from various lakes and long-time surveys on the relationship of environment-rotifer communities based on their taxon- and trait-based indices are also needed to better ascertain how rotifer communities respond to changes in environmental conditions.

## Electronic supplementary material

Supplementary information
