# Supplementary material for: Comparative analysis of the spatio-temporal dynamics of rotifer community structure based on taxonomic indices and functional groups in two subtropical lakes
Source: Sci Rep. 2017 Apr 3;7:578. doi: 10.1038/s41598-017-00666-y (PMC5428772; doi:10.1038/s41598-017-00666-y)
Supplement: Supplementary file 1 — Supplementary information [file 41598_2017_666_MOESM1_ESM.pdf]

Comparative analysis of the spatio-temporal dynamics of rotifer community structure based on taxonomic indices and functional groups in two subtropical lakes

Xinli Wen[1,2*], Pan Zhai[1], Ruonan Feng[1], Ruijie Yang[1], Yilong Xi[1,2]

[1] *Key Laboratory of Biotic Environment and Ecological Safety in Anhui Province; College of Life Sciences, Anhui Normal University, Wuhu 241000, China*

[2] *Collaborative Innovation Center of Recovery and Reconstruction of Degraded Ecosystem in Wanjiang City Belt, Anhui Province, Wuhu 241000, China*

Correspondence and requests for materials should be addressed to X.L.W. (email: wenxinli1977@126.com)

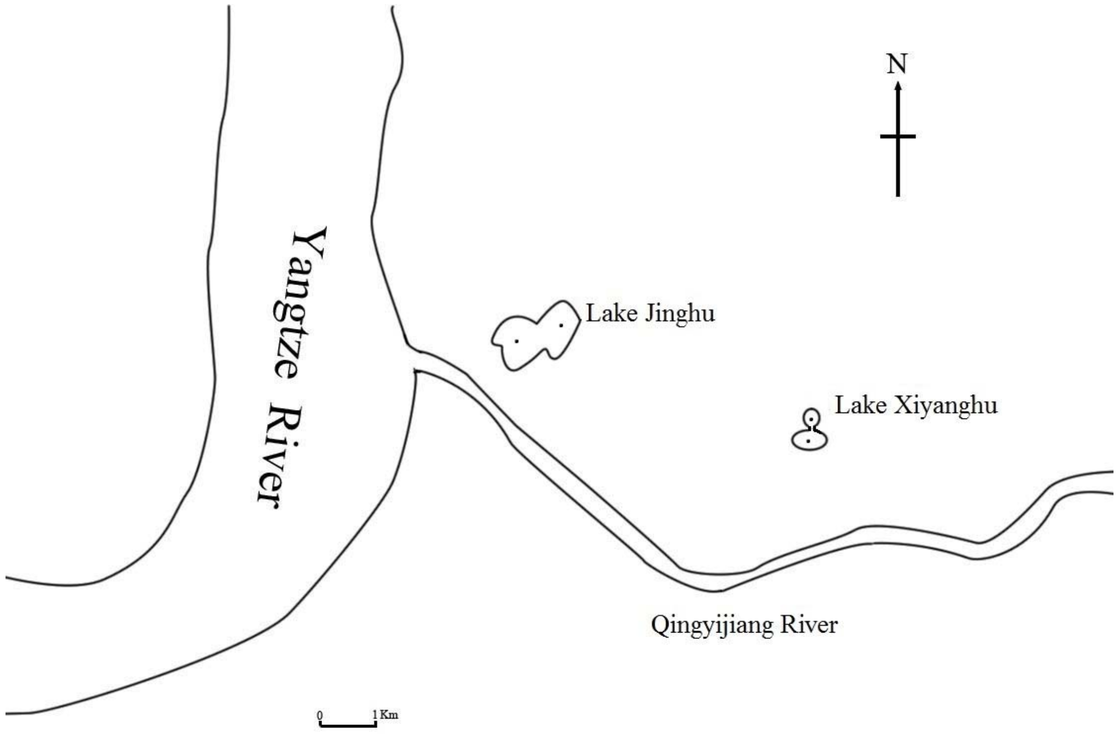

Supplementary Figure S1. Sampling locations for rotifers in Lake Jinghu and Lake Xiyanghu. This map was drawn by hand using Photoshop (version CS6, http://www.adobe.com/cn/products/cs6/photoshop.html) based the picture from Google Earth (version 7.1, http://www.earth.google.com).

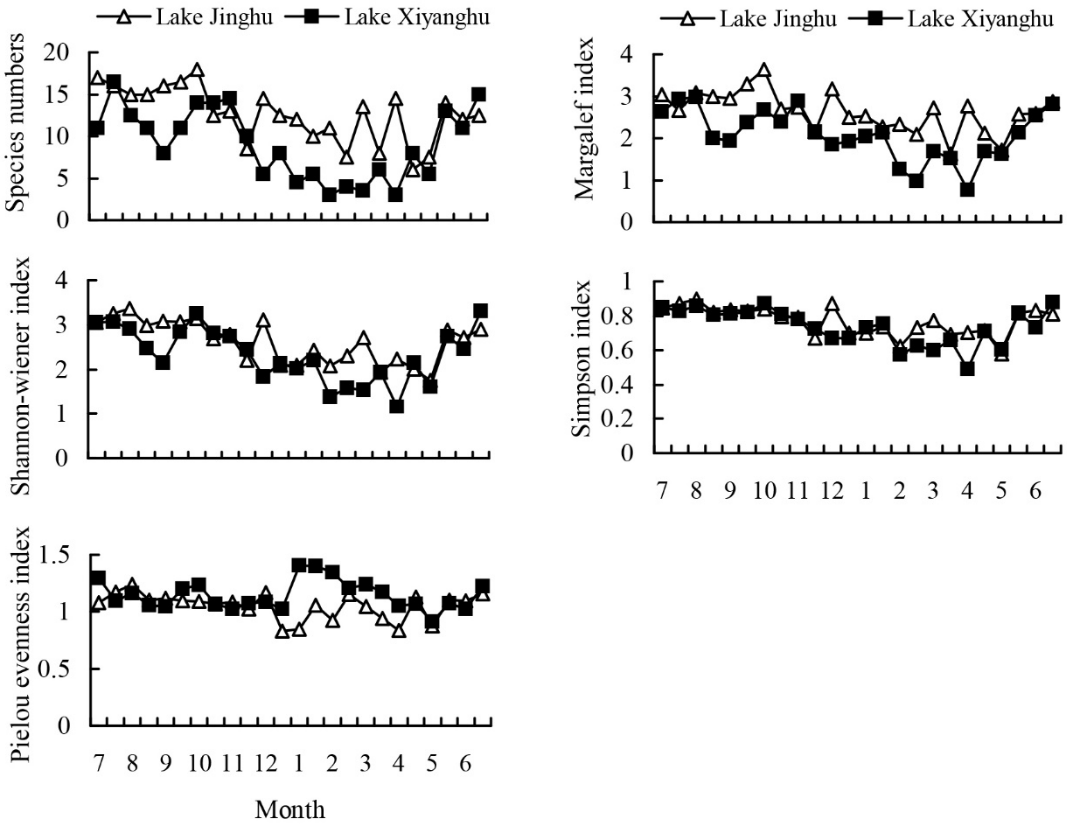

Supplementary Figure S2. Annual dynamics of taxon-based indices of rotifer communities, including Species numbers, Margalef index, Simpson index, Shannon-wiener index, and Pielou evenness index.

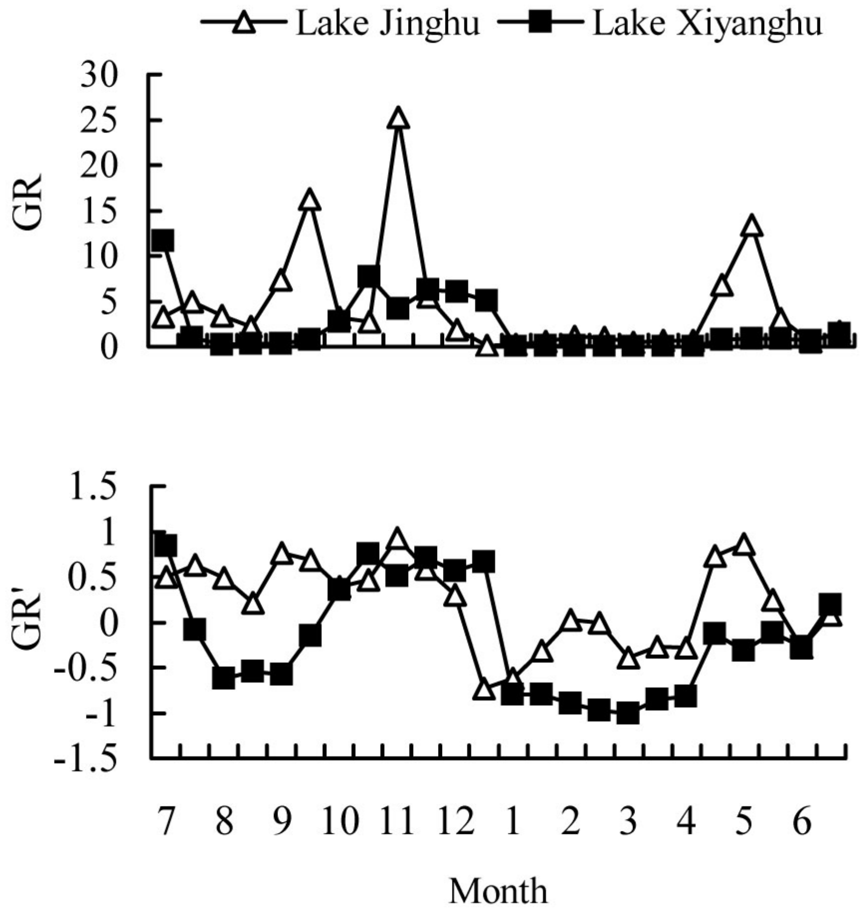

Supplementary Figure S3. Annual dynamics of trait-based indices of GR and GR’ in rotifer communities.

Supplementary Table S1. Species composition of rotifers.

Species	Lake Jinghu		Lake Xiyanghu	
	Station 1	Station 2	Station 1	Station 2
**Brachionidae**				
Brachionus calyciflorus	+	+	+	+
B. diversicornis	+	+	+	+
B. forficula	+	+	+	+
B. angularis	+	+	+	+
B. urceolaris	+	+	+	+
B. budapestinensis	+	+	+	+
B. quadridentatus			+	
Keratella cochlearis	+	+	+	+
K. valga			+	+
Anuraeopsis fissa	+	+	+	+
A. navicula	+	+	+	+
Notholca labis	+	+	+	+
**Filiniidae**				
Filinia longiseta	+	+	+	+
F. terminalis	+	+		+
F. minuta	+	+	+	+
F. cornuta	+	+	+	+
F. brachiata			+	+
**Asplanchnidae**				
Asplanchna priodonta	+	+	+	+
A. girodi	+	+	+	+
A. brightwelli		+	+	+
Asplanchna sp.			+	+
Asplanchnopus multiceps	+	+		
A. hyalinus	+	+		
**Trichocercidae**				
Trichocerca pusilla	+	+	+	+
T. capucina	+	+		
T. longiseta	+	+		
T. similes	+	+		
T. tenuior			+	+
T. cylindrica			+	+
T. rousseleti			+	+
T. elongata			+	+
T. rattus	+	+		
Trichocerca sp.	+			+
**Synchaetidae**				
Synchaeta oblonga	+	+	+	+

Table S1 *continued*

S. stylata	+	+		
S. pectinata	+	+	+	+
Synchaeta sp.			+	+
Polyarthra vulgaris	+	+	+	+
**Gastropodidae**				
Gastropus minor	+	+		
G. hyptopus	+	+	+	+
Ascomorpha ecaudis	+	+	+	
A. saltans	+	+		+
**Hexarthridae**				
Hexarthra mira	+	+	+	
**Lecanidae**				
Lecane pyriformis	+			
L. lunaris	+	+		+
L. cornuta		+		
Lecane sp.			+	+
**Dicranophoridae**				
Dicranophorus uncinatus			+	+
**Conochilidae**				
Conochilus unicornis	+	+		
**Collothecidae**				
Collotheca ornata	+	+		
**Euchlanidae**				
Euchlanis triquetra	+			
**Notommatidae**				
Notommata aurita			+	
N. silpha			+	+
N. cyrtopus		+		
Cephalodella Megalocephala		+		+
C. exigua			+	+
C. incila			+	
C. gibba			+	+
**Testudinellidae**				
Testudinella patina				+
**Epiphanidae**				
Rhinoglena frontalis	+	+		
**Proalidae**				
Proales parasitica		+	+	+
**Philodinidea**				
Rotaria rotatoria	+	+	+	+
R. tardigrada			+	+
R. neptunia	+	+		+
